# Breast cancer screening beliefs questionnaire: psychometric properties of the Persian version

**DOI:** 10.1186/s12905-020-01049-8

**Published:** 2020-08-17

**Authors:** Hamid Sharif Nia, Fereshteh Behmanesh, Cannas Kwok, Mojgan Firouzbakht, Abbas Ebadi, Maryam Nikpour

**Affiliations:** 1 School of Nursing and Midwifery Amol, Mazandaran University of Medical Sciences, Sari, IR Iran; 2 Social Determinants of Health Research Center, Department of Midwifery, School of Medicine, Babol University of Medical Sciences, Babol, IR Iran; 3 Sydney Nursing School, School of Nursing and Midwifery, Western Sydney University, Sydney, New South Wales Australia; 4 Reproductive health, Student Research Committee, Health Research Institute, Babol. University of Medical Sciences, Babol, IR Iran; 5 Behavioral Sciences Research Center, Nursing Faculty, Life style institute, Baqiyatallah University of Medical Sciences, Tehran, IR Iran; 6 Department Nursing and Midwifery, Faculty of Medical Sciences, Islamic Azad University, Babol branch, Babol, Iran; 7 Student Research Committee, Health Research Institute, Babol. University of Medical Sciences, Babol, IR Iran

**Keywords:** Psychometric evaluation, Breast cancer screening, Validity, Reliability, Iran, Women

## Abstract

**Background:**

Valid and reliable instruments are needed to assess such beliefs, attitudes, and knowledge. This study aimed to translate Breast Cancer Screening Beliefs Questionnaire into Persian and evaluate its psychometric properties among Iranian women.

**Methods:**

In this methodological study, the twelve-item Breast Cancer Screening Beliefs Questionnaire was translated into Persian and filled out by 1256 Iranian women. Face, content, convergent, and discriminant validity were evaluated and exploratory and confirmatory factor analyses were performed for construct validity evaluation. Reliability was also evaluated through calculating Cronbach’s alpha, McDonald’s omega, Average inter-item correlation, and test-retest intraclass correlation coefficient and finally, composite reliability was estimated.

**Results:**

Three factors were extracted in factor analysis which included screening attitude, screening knowledge and perception, and screening practice. These factors explained 55.71% of the total variance of breast cancer screening beliefs. This three-factor model was confirmed in confirmatory factor analysis based on model fit indices (PCFI = 0.703, PNFI = 0.697, CMIN/DF = 2.127, RMSEA = 0.30, GFI = 0.980, AGFI = 0.998, and CFI = 0.991). Convergent and discriminant validity were also confirmed. Composite reliability and test-retest intraclass correlation coefficient were more than 0.7.

**Conclusion:**

With a three-factor structure, the Persian Breast Cancer Screening Beliefs Questionnaire has acceptable validity and reliability and hence, can be used to evaluate Iranian women’s breast cancer screening beliefs.

## Background

Breast cancer (BC) is the most prevalent malignancy [1, 2] and the second leading cause of death after lung cancer [3] among women worldwide. It is among the most costly cancers in the world with the annual financial burden of 88 billion dollars. Its annual cost per afflicted woman is estimated to be around 1.5 million dollars [4]. BC is also the most prevalent cancer among Iranian women and accounts for 25.4% of all female malignancies [5]. Besides, the age of affliction by BC in Iran is around 10 years lower than other developed countries [6].

Delay in cancer diagnosis is a major factor behind its high mortality rate because survival is directly associated with the stage of cancer at diagnosis [7]. The five-year survival rate of BC in developed countries such as the United States and England is 85–95% [8]. However, two third of Iranian women with BC experience early death due to the delays in the diagnosis of BC [9].

The major reasons behind delayed diagnosis of BC in Iran may be women’s lack of knowledge, delays in seeking medical help, and failure to participate in BC screening programs [10] such as breast self-examination, periodical medical visits, and mammography [11]. Women’s participation in screening programs is affected by different factors, chiefly their health beliefs [12] and health-related knowledge [13], so that positive health beliefs and adequate health-related knowledge can increase participation in such programs [13]. In western countries, people have adequate health-related knowledge and positive health beliefs; thus, periodical health assessment in the absence of any health problem is a known concept to the public and a routine practice [14]. However, this is an unfamiliar concept for people in most Asian countries [15]. Qualitative studies in Iran showed that due to cultural reasons, Iranian women are inattentive to and neglectful of their health [3] and have misconceptions about BC [16]. Accordingly, educational interventions on BC can positively affect women’s screening-related behaviors [17] and thereby, contribute to early cancer diagnosis and improvements in quality of life and survival [18].

Accurate assessment of women’s beliefs about BC screening requires valid and reliable instruments. Such instruments help generate more reliable and conclusive results and develop more effective BC screening and prevention programs [19]. However, previous studies on Iranian women’s knowledge, attitudes, and practice respecting BC screening used instruments that their validity and reliability had not been evaluated using standard methods [20]. Moreover, some studies used the Champion’s Health Belief Model Scale which has limitations such as large number of items (57 items) [21].

Breast Cancer Screening Beliefs Questionnaire (BCSBQ) is a short twelve-item instrument for the assessment of BC screening beliefs. It is easy to use and has appropriate scoring system and high sensitivity; hence, it is considered a good instrument for the assessment of BC screening beliefs [22]. However, it has no valid and reliable Persian version. Thus, the present study was conducted to translate BCSBQ into Persian and evaluate its psychometric properties among Iranian women.

## Methods

This cross-sectional methodological study was carried out in 2017–2018.

### Sample

There is no universal consensus over sampling adequacy in psychometric studies. However, samples greater than 1000 are considered adequate [23]. Therefore, sample sizes for exploratory and confirmatory factor analyses in the present study were considered to be 800 and 500, respectively. Sampling was purposively done from June 2017 to March 2018 in three central cities in Mazandaran province, Iran, namely Amol, Babol, and Sari. Inclusion criteria were ability to read and write in Persian, an age of more than 18 and no history of BC among family members (i.e. mother, sister, or daughter).

### Instrument

The instrument of the study was BCSBQ developed by Kwok et al. in 2010. BCSBQ has 12 items on women’s attitudes towards general health assessment, their knowledge, attitudes, and perceptions regarding BC, and their screening practice in the area of mammography. BCSBQ items are scored using a Likert-type scale from 1 (“Completely agree”) to 5 (“Completely disagree”). The total score of the questionnaire is changed into a 0–100 scale. The developers of the questionnaire found that it has three subscales and reported a Cronbach’s alpha of 0.84 for it [22].

### Translation

After obtaining necessary permissions for using BCSBQ from professor Kwok, the questionnaire was translated into Persian based on the forward-backward translation protocol proposed by the World Health Organization [24]. Initially, a reproductive health specialist and an English expert independently translated the questionnaire into Persian and then, the authors developed a single Persian translation of BCSBQ based on their translations. After that, two other translators (a reproductive health specialist and an English expert) independently back-translated the final Persian version of the questionnaire into English. The authors used these two English translations to develop a single English translation. Finally, the final English translation was sent to professor Kwok for the purpose of approval. She approved that our English BCSBQ was similar to her original questionnaire.

#### Psychometric evaluation

##### Face validity evaluation

Twenty women were provided with the Persian BCSBQ and were asked to assess the clarity and simplicity of its items. None of them reported ambiguities in BCSBQ items.

##### Content validity evaluation

Content validity was evaluated through qualitative and quantitative methods [25]. In qualitative content validity evaluation, the questionnaire was given to 10 experts in instrument development and healthcare (six reproductive health specialists with PhD degree, one health education specialist with master’s degree, two midwives with master’s degree, and one clinical psychologist with PhD degree).. Qualitative content validity of the questionnaire was approved after making revisions recommended by the specialists. They were asked to evaluate appropriate wording and placement of the items. They recommended some linguistic amendments to the questionnaire items. Quantitative content validity evaluation was performed through calculating content validity ratio (CVR) and content validity index (CVI) for the questionnaire. For CVR calculation, the aforementioned 10 specialists rated the essentiality of BCSBQ items as “Essential” (scored 1), “Not essential, but useful” (scored 2), and “Not essential” (scored 3). Items which were considered essential by nine specialists were kept. Among 10 specialists, nine determined that all items were essential and therefore, no item was removed. For CVI calculation, the specialists were asked to rate the relevance of the items on the following scale: 1: “Irrelevant”; 2: “Somewhat relevant”; 3: “Acceptably relevant”; 4: “Completely relevant”. Subsequently, CVI of each item was calculated through dividing the number of specialists who had rated that item 3 or 4 by 10. CVI values of 0.78 and more were considered acceptable [26]Moreover, quantitative content validity evaluation showed that all items had CVRs greater than 0.8 and CVIs greater than 0.78. Therefore, none of the items were removed.

##### Construct validity evaluation

Construct validity was evaluated through with exploratory and confirmatory factor analysis. Exploratory factor analysis (EFA) is a multivariate statistical technique that describer the relationship of some observed variables by a relatively number of factors [27] Initially, maximum likelihood EFA with varimax rotation was performed. Kaiser-Meyer-Olkin (KMO) test was run to determine sample adequacy, while Bartlett’s test was run to evaluate homogeneity of variance. Then, latent factors were extracted based on Horn’s Parallel Analysis, and scree plot [28]. According to the three-indicator rule, each factor had to have at least three items. All these analyses were performed in SPSS_25_, SPSS R-Menu_2_ JASP_0.9.0.1_. After EFA, confirmatory factor analysis (CFA) with maximum likelihood estimation was performed using the AMOS_24_ software to test the fit of the extracted model based on the most commonly used indices for model fit. CFA state the degree of disharmony, between predicted and empirical factor structure in χ2 and indices [29]. These indices were Parsimonious Comparative Fit Index (PCFI), Parsimonious Normed Fit Index (PNFI), Minimum Discrepancy Function divided by Degrees of Freedom (CMIN/DF), Root Mean Square Error of Approximation (RMSEA), Adjusted Goodness of Fit Index (AGFI), and Comparative Fit Index (CFI).

##### Convergent and discriminant validity evaluation

Based on Fornell and Larcker’s criteria [30], convergent and discriminant validity and construct reliability were evaluated through calculating Average Variance Extracted (AVE), Maximum Shared Squared Variance (MSV), Average Shared Squared Variance (ASV), and Composite Reliability (CR). In order to confirm convergent validity, AVE should be greater than 0.5 and CR should be greater than AVE. On the other hand, to ensure discriminant validity, AVE should be greater than MSV [31]. Moreover, a scale has acceptable convergent validity when all its items are close together and share a large amount of variance, while it has acceptable discriminant validity when the extracted factors are completely independent from each other [32]. Convergent and discriminant validity evaluations revealed that all factors had acceptable convergent and discriminant validity.

##### Relative reliability evaluation

Relative reliability was evaluated through the test-retest method, in which 12 participants filled out BCSBQ twice with a two-week period in between. Then, intraclass correlation coefficient (ICC) was calculated using two-way mixed effects model. Moreover, Cronbach’s alpha, McDonald’s omega, and Average inter-item correlation were calculated for internal consistency evaluation [31]. Internal consistency assesses item homogeneity, or the degree to which the items on a test jointly measure the same construct [33]. Then, construct reliability (CR) was evaluated. CR value greater than 0.7 was considered as acceptable reliability [34].

##### Absolute reliability evaluation

ICC provides no accurate information about the accuracy of the scores. Therefore, absolute reliability was estimated by calculating standard error of measurement (SEM) using the following formula, $SEM= SD\sqrt{1- ICC}$ (37).

##### Ethics consideration

This study is approved by the Ethics Committee of Health Research Institute in Babol University of Medical Sciences. [Grant number: MUBABOL, HRI.REC.1396.10].

## Results

In total, 1300 women were recruited to fill out BCSBQ, 1256 of them completely filled out and returned their questionnaires (response rate: 96%). The median of women’s age was 32 (IQR 27, 39). Most women were married (87.5%) and lived in urban areas (65.7%). More than one third of them had university degrees and was employed (Table 1).

**Table 1:** Participants’ demographic characteristics

Characteristics		Median	Interquartile range	Total
**Age (Years)**		32	27,39	1047
**Age at menarche (Years)**		13	12,14	1010
**Characteristics**		**N**	**%**	**Total**
**Educational status**	Below diploma	343	29	1183
	Diploma	392	33.1	
	University	448	37.9	
**Place of residence**	Urban areas	763	65.7	1162
	Rural areas	339	34.3	
**Employment status**	Housewife	773	67	1155
	Employed	294	25.4	
	Student	88	7.6	
**Marital status**	Single	117	10	1169
	Married	1023	87.7	
	Widowed	29	2.5	
**Income level**	High	234	27.3	1185
	Moderate	742	62.7	
	Low	119	10	
**Number of children**	0	183	18.2	1008
	1	340	27.1	
	2	485	38.6	
	≥ 3	433	43.5	

In exploratory factor analysis, KMO test value was 0.78 and Bartlett’s test value was 3349.82 (*P* <  0.001). Three factors were extracted and named as *screening attitude, screening knowledge and perception, and screening practice*. These three factors explained 55.71% of the total variance of BC screening beliefs (Table 2).

**Table 2:** The three factors of the Persian BCSBQ and their items

Factors	Items	Factor loading	Item communality	Variance	Eigen value
**Screening attitude**	Q_4_. *I do not see a doctor when I am healthy*.	0.78	0.61	22.81	2.51
	Q_3_. *I see a doctor or get a checkup whenever I have a health problem*.	0.76	0.57		
	Q_1_. *I do not need any checkups when I feel good.*	0.75	0.55		
	Q_2_. *I do not need any checkups when I have a healthy lifestyle, a balanced diet and regular fitness activities.*	0.72	0.52		
**Screening knowledge and perception**	Q_6_. *Breast cannot be cured, the only thing you can do is to prolong the suffering period.*	0.79	0.62	17.36	1.97
	Q_5_. *Breast cancer is lethal and if you get breast cancer you will certainly die.*	0.56	0.29		
	Q_8_. *If a woman’s fate is to get breast cancer, she will and she can do nothing to change her fate.*	0.55	0.33		
	Q_7_. *Even if breast cancer is diagnosed in its early stages, there is very little chance of survival for the patient.*	0.53	0.23		
**Screening practice**	Q_12_. *Mammography makes me feel shamed and embarrassed.*	0.83	0.68	15.54	1.71
	Q_10_. *It is hard for me to commute for mammography.*	0.52	0.30		
	Q_11_. *I do not want to get a mammography because I have to take of my clothes and expose my breasts.*	0.49	0.23		

**Fig. 1:**
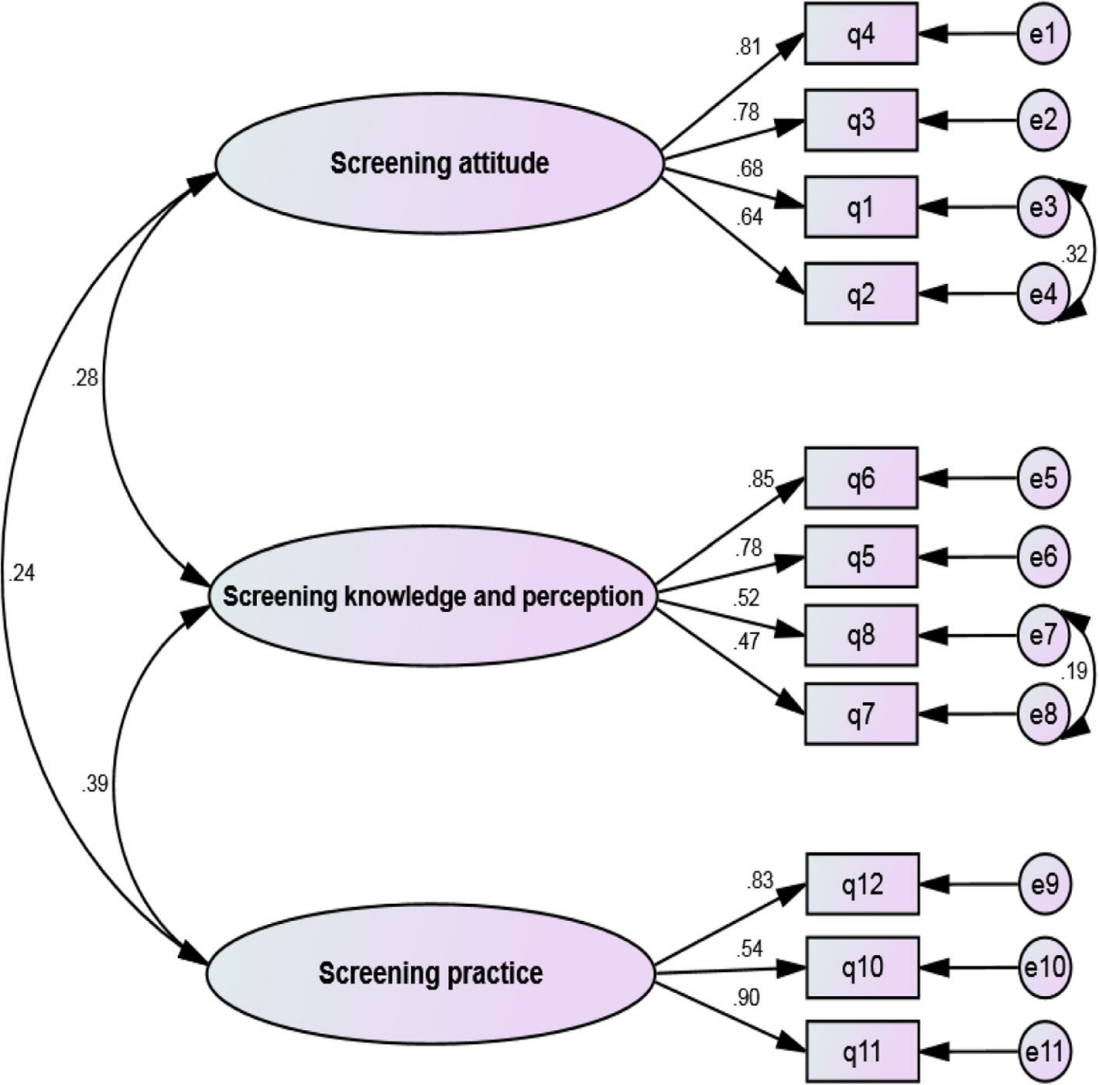
The final factor analysis model for BCSBQ

In confirmatory factor analysis, after correcting the model, the Chi-square model fit index was calculated which was equal to 82.93 (*P* <  0.001). The model was corrected through drawing the correlations between the measurement errors of items 1 and 2 (e3 and e4) and between the measurement errors of items 7 and 8 (e7 and e8), (Fig. 1). Then, other model fit indices were calculated as the following, PCFI = 0.703, PNFI = 0.697, CMIN/DF = 2.127, RMSEA = 0.30, GFI = 0.980, AGFI = 0.998, and CFI = 0.991. These values confirmed the good fit of the final model (Table 3).

**Table 3:** The model fit indices in confirmatory factor analysis

Indices	χ^**2**^	df	***P*** value	CMIN/DF	RMSEA	PCFI	PNFI	AGFI	GFI	CFI
Values after correction	82.93	39	< 0.001	2.127	0.030	0.703	0.697	0.980	0.998	**0.991**

*DF* Degree of freedom, *PCFI* Parsimonious Comparative Fit Index, *PNFI* Parsimonious Normed Fit Index, *CMIN/DF* Minimum Discrepancy Function divided by Degrees of Freedom, *RMSEA* Root Mean Square Error of Approximation, *AGFI* Adjusted Goodness of Fit Index, and *CFI* Comparative Fit Index

Internal consistency evaluation revealed that the Cronbach’s alpha, McDonald’s omega, and Average inter-item correlation were greater than 0.7 and 0.4 respectively. Moreover, CR was more than 0.75 and ICC was more than 0.7 (Table 4). SEM was estimated to be ±2.14.

**Table 4:** The indices of the convergent and discriminant validity, internal consistency, and stability of BCSBQ

Indices	ASV	MSV	AVE	CR	Alpha (CI95%)	AIC	Omega
Factors							
**Screening attitude**	0.069	0.078	0.537	0.821	0.836 (0.820 to 0.850)	0.560	0.836
**Screening knowledge and perception**	0.117	0.156	0.452	0.757	0.745 (0.721 to 0.768)	0.438	0.766
**Screening practice**	0.108	0.156	0.594	0.808	0.786 (0.765 to 0.806)	0.556	0.808

*ASV* Average Shared Squared Variance, *MSV* Maximum Shared Squared Variance, *AVE* Average Variance Extracted, *CR* Composite Reliability, *Alpha* Cronbach’s alpha, *AIC* Average Inter-item Correlation, *Omega* McDonald’s omega coefficient

## Discussion

This study aimed to translate BCSBQ into Persian and evaluate its psychometric properties among Iranian women. Findings revealed a three-factor structure for the questionnaire which explained 55.71% of the total variance of BC screening beliefs. Our findings denotes the appropriateness of the questionnaire for assessing BC screening beliefs among Iranian women because an explained variance of more than 50% is indicative of the appropriateness of the extracted factors [35]. In line with the findings of the present study, previous studies on Arab, Chinese Australian [14, 22], Indian Australian [36], African Australian [37], and Korean women [38] also reported that the questionnaire had three factors.

Screening attitude was the first extracted factor of BCSBQ in the present study. The four items of this factor had high correlation with the factor. This factor deals with Iranian women’s attitudes towards general health screening. In other words, it assesses women’s attitudes towards the necessity of periodical health assessment despite feeling healthy. This factor seems to be in line with the perceived susceptibility construct of the Health Belief Model. The model is used to assess people’s beliefs about screening behaviors [39]. The perceived susceptibility construct of this model refers to person’s beliefs about the risk or the chance of developing a disease such as BC [39].

The second factor of the Persian BCSBQ was screening knowledge and perception. This factor includes four items on knowledge and perceptions about screening. The multi-component PEN-3 (Person, Extended family, and Neighbor) model also includes a perception component. Perception in that model encompasses knowledge, beliefs, and values which can enhance or reduce motivation for behavioral modification [40]. The items of the knowledge and perception domain of the Persian BCSBQ deal with women’s knowledge and perception about the probability of reducing BC complications or postponing BC-induced death through appropriate screening. Accurate assessment of knowledge and perception can help develop effective interventions for health promotion [41].

Study findings revealed significant correlations between the measurement errors of items 1 and 2 (e3 and 34) and between the measurement errors of items 7 and 8 (e7 and e8). Measurement error happens when items are not well understood or are not directly measured or happens due to the conceptual similarity of two items or words [42]. Items 1 and 7 convey almost the same meaning as respectively items 2 and 8; thus, correlations between the measurement errors of items 1 and 2 and between the measurement errors of items 7 and 8 are justifiable.

Screening practice was the third factor of the Persian BCSBQ. This factor assesses mammography-related behavior and its barriers. Behavior is one of the most important components of screening programs. In other words, the behavior dimension of these programs assesses whether knowledge improvement and attitude change have been effective in modifying screening behavior [43]. After assessing knowledge and attitudes in the first and the second dimensions, the screening practice dimension of BCSBQ assesses women’s mammography-related behavior, which is the most important BC screening behavior.

Cronbach’s alpha, McDonald’s omega, AIC, test-retest ICC, and CR values revealed that the Persian BCSBQ has acceptable reliability. Previous studies on Arab, Indian Australian, and African Australian women also showed a Cronbach’s alpha of more than 0.8 for BCSBQ [14, 22, 36–38]. AIC of the factors were greater than 0.4. The AIC of the items should be ranged between 0.2–0.4, while ideals in the range 0.1–0.5 are acceptable [44]. The AIC for the three sub-scales were greater than 0.4, respectively, thus demonstrating reasonable reliabilities. This study had two limitations. The study was imprecise answering to BCSBQ items by some participants as well as their sociocultural wide diversity. Second, the sampling was done in three central parts of Mazandaran province, if it was done in more cities, generalizability.

The Persian BCSBQ can be used in healthcare centers and gynecology clinics to assess Iranian women’s beliefs about BC screening. The results of such assessment can help develop and use educational and counseling interventions for correcting women’s misconceptions and improving their knowledge about BC screening. One of the most important factors in improving health behavior screening is positive health beliefs. Developed countries such as the United States, etc., were successful in reduce the mortality rate from breast cancer by positive health beliefs and raising health-related knowledge.

## Conclusion

The Persian BCSBQ has acceptable factor structure and internal consistency. Therefore, it can be used as a valid and reliable tool for assessing BC screening beliefs among Iranian women.

### Implications for clinical practice

The Persian BCSBQ can be used in healthcare centers and gynecology clinics to assess Iranian women’s beliefs about BC screening. The results of such assessment can help develop and use educational and counseling interventions for correcting women’s misconceptions and improving their knowledge about BC screening.

## Data Availability

The data are available from the corresponding author on reasonable request.

## Abbreviations

BC: Breast Cancer
BCSBQ: Breast Cancer Screening Beliefs Questionnaire
CVR: Content Validity Ratio
CVI: Content Validity Index
EFA: Exploratory factor analysis
CFA: Confirmatory factor analysis
AVE: Average Variance Extracted
MSV: Maximum Shared Squared Variance
ASV: Average Shared Squared Variance
CR: Composite Reliability
AVE: Confirm Convergent Validity
AMOS: Analysis of Moment Structures
EDA: Exploratory Data Analysis
ICC: Intraclass Correlation Coefficient
CR: Construct Reliability
SEM: Standard Error of Measurement
